# Triggering Pyroptosis in Cancer

**DOI:** 10.3390/biom15030348

**Published:** 2025-02-28

**Authors:** Daniel E. Johnson, Zhibin Cui

**Affiliations:** 1 Department of Otolaryngology—Head and Neck Surgery, University of California at San Francisco, San Francisco, CA 94143, USA; daniel.johnson@ucsf.edu; 2 Helen Diller Family Comprehensive Cancer Center, University of California at San Francisco, San Francisco, CA 94143, USA; 3 Department of Oral Biology, School of Dental Medicine, University at Buffalo, The State University of New York, Buffalo, NY 14214, USA

**Keywords:** cancer, pyroptosis, cell death, chemotherapy drugs, caspase-1, gasdermin, IL-1b, IL-18, *GSDM-E*, natural products, LPS

## Abstract

Pyroptosis is an inflammatory programmed cell death recently identified as a crucial cellular process in various diseases, including cancers. Unlike other forms of cell death, canonical pyroptosis involves the specific cleavage of gasdermin by caspase-1, resulting in cell membrane damage and the release of the pro-inflammatory cytokines IL-1β and IL-18. Initially observed in innate immune cells responding to external pathogens or internal death signals, pyroptotic cell death has now been observed in numerous cell types. Recent studies have extensively explored different ways to trigger pyroptotic cell death in solid tumors, presenting a promising avenue for cancer treatment. This review outlines the mechanisms of both canonical and noncanonical pyroptosis pertinent to cancer and primarily focuses on various biomolecules that can induce pyroptosis in malignancies. This strategy aims not only to eliminate cancer cells but also to promote an improved tumor immune microenvironment. Furthermore, emerging research indicates that targeting pyroptotic pathways may improve the effectiveness of existing cancer treatments, making them more potent against resistant tumor types, offering new hope for overcoming treatment resistance in aggressive malignancies.

## 1. Introduction to Pyroptosis

Homeostasis of cell numbers in eukaryotes is maintained via a balance between cellular proliferation and cell death pathways. In cancer, this balance is lost, either as a result of aberrant proliferation or the loss of normal cell death processes, or both. Multiple forms of cell death have been described, with apoptosis being the most extensively characterized. Numerous studies have shown that apoptotic cell death is frequently abrogated in human tumors, resulting in tumor growth and resistance to anti-cancer drugs and biologics. More recently, additional forms of cell death have been identified, including autophagy, necroptosis, pyroptosis, ferroptosis, cuproptosis, and disulfidptosis. In this review, we summarize the current state of knowledge regarding the mechanisms of pyroptosis and the molecules and drugs that can be used to induce this cell death process.

The term pyroptosis was introduced by Cookson and Brennan in 2001 to describe a novel form of cell death distinct from apoptosis [1]. While the hallmark cell death features of nuclear condensation and DNA fragmentation are observed in both pyroptosis and apoptosis, the process of pyroptosis is uniquely dependent on caspase-1 protease, whereas apoptosis is typically dependent on the caspase-3 or caspase-7 proteases [1,2,3]. Caspase-1 is responsible for proteolytic production of the pro-inflammatory cytokines interleukin-1β (IL-1β) and interleukin-18 (IL-18) [4]. Hence, in contrast to apoptosis, which does not induce inflammation, cell death resulting from pyroptosis is pro-inflammatory. Moreover, pyroptosis is associated with rapid damage to the cell membrane, leading to the early release of pro-inflammatory cytokines [3]. This also contrasts with apoptotic cell death, where the loss of cell membrane integrity is a late event in the death process. Collectively, pyroptosis is distinguished from apoptosis by caspase-1 dependency, pro-inflammatory cytokine production, and early cell membrane rupture.

Pyroptosis was initially described as a cellular suicide process that macrophages undergo in response to interaction with Salmonella or other pathogens [2,5,6]. Later, the process was identified in other innate immune cells, including neutrophils and the barrier intestinal epithelial cells [7]. The induction of pyroptosis during an innate immune response is believed to function in fighting infections by promoting the release of pro-inflammatory cytokines (i.e., IL-1β and IL-18), resulting in the attraction of other immune cells to the site of infection and effective pathogen elimination. However, due to its inflammatory-associated properties, pyroptosis has also been found to be associated with several chronic inflammatory diseases, such as arthritis, pneumonia, hepatitis, colitis, and cardiovascular disease [8,9]. In addition, significant associations have been reported between pyroptosis and various cancers, highlighting the prospect of inducing this unique form of cell death as a novel therapeutic approach for cancer. In this review, we discuss the role of pyroptosis in cancer and explore promising biomolecules that might be utilized to induce pyroptosis in human malignancies.

## 2. Pyroptosis Signaling Pathways

Pyroptosis is characterized by rapid cell membrane rupture, driven by the gasdermin (GSDM) protein family (particularly GSDM-D), along with secretion of the pro-inflammatory cytokines IL-1β and IL-18, which recruit other innate immune cells and enhance the cytolytic activities of NK and T-cells [10,11]. Initially, this process was thought to be mediated specifically by caspase-1, defining what is now known as the canonical pathway. However, in the past decade, additional mechanisms of pyroptosis activation have been identified, leading to the classification of these alternative routes as noncanonical pathways.

### 2.1. Canonical Pathway of Pyroptosis

**Figure 1:**
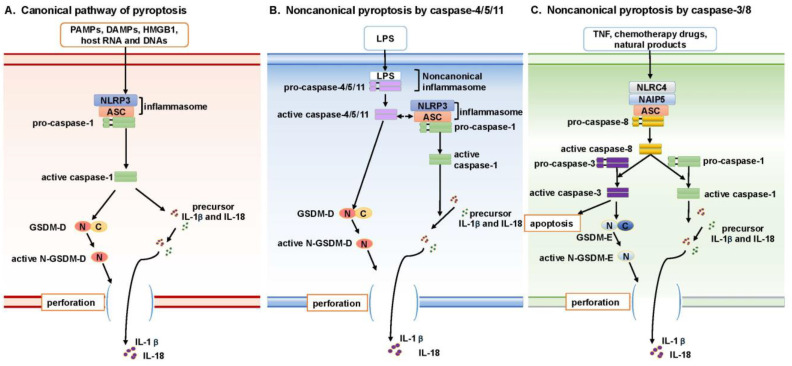
Canonical and noncanonical pyroptosis signaling pathways. (**A**) The canonical pyroptosis pathway in innate immune cells, like macrophages, is initiated by PAMPs and DAMPs that activate pattern recognition receptors (PRRs), primarily the NLRP3 receptor. When NLRP3 is engaged, it forms the inflammasome complex with ASC, which subsequently recruits and activates caspase-1. The active form of caspase-1 cleaves GSDM-D, leading to the formation of active N-terminal fragments (N-GSDM-D) that create pores in the cell membrane, culminating in pyroptotic cell death. Additionally, caspase-1 cleaves and activates the pro-inflammatory cytokines IL-1β and IL-18, which are released upon cell rupture and serve to attract and activate both innate and adaptive immune cells, thereby promoting inflammation. (**B**) Noncanonical pyroptosis pathways operate independently of the NLRP3/ASC/caspase-1 axis and involve the direct detection of LPS by caspase-11 in mice or by caspase-4/5 in humans. This engagement leads to the formation of a “noncanonical inflammasome”, activating these caspases and resulting in GSDM-D cleavage and pyroptotic cell death. While caspase-11 does not directly process IL-1β or IL-18, it can activate the canonical NLRP3/caspase-1 inflammasome to enhance cytokine release. (**C**) Apoptotic mediators such as caspase-3 and caspase-8 can also provoke pyroptosis via noncanonical pathways triggered by TNF, chemotherapy agents, or natural products. Caspase-3 cleaves GSDM-E into N-terminal fragments, and caspase-8 can induce pyroptosis in macrophages that lack caspase-1 and -11 through a GSDM-D-independent mechanism that involves the NAIP5/NLRC4/ASC inflammasome.

The canonical pyroptosis pathway (Figure 1A) was originally identified in innate immune cells or antigen-presenting cells, such as macrophages and monocytes [12,13]. These cells play a defensive role by detecting “danger” signals—including pathogens, cell stress, cytokines, or disruptions to cellular homeostasis—which activate pyroptotic cell death and trigger inflammation [14].

During infection, microorganisms release pathogen-associated molecular patterns (PAMPs) like lipopolysaccharide (LPS), while stressed or damaged host cells release damage-associated molecular patterns (DAMPs), including histones, hyaluronic acid, high-mobility group protein B1 (HMGB1), and fragments of host cell RNA and DNA [15]. PAMPs and DAMPs are recognized by pattern recognition receptors (PRRs), leading to the activation of immune cells. Based on homologies in protein domains, several different PRRs have been defined, including Toll-like receptors (TLRs), nucleotide oligomerization domain (NOD)-like receptors (NLRs), retinoic acid-inducible gene-I (RIG-I)-like receptors (RLRs), C-type lectin receptors (CLRs), and absent in melanoma-2 (AIM2)-like receptors (ALRs). The most extensively studied PRR involved in the canonical pyroptosis pathway is NLRP3 (NOD-, LRR-, and pyrin domain-containing protein 3) [16,17]. Activated NLRP3 induces the assembly of the inflammasome complex by recruiting pro-caspase-1 and the adapter protein ASC (apoptosis-associated speck-like protein containing a caspase recruitment domain). The inflammasome complex recruits multiple pro-caspase-1 molecules, resulting in processing to generate active caspase-1. The activated caspase-1 then cleaves the substrate protein gasdermin D (GSDM-D), generating proteins encompassing the N-terminal domain (N-GSDM-D) and the C-terminal domain (C-GSDM-D). The N-GSDM-D protein is recruited to the cell membrane where it forms pores, resulting in the early rupture of the cell membrane, a defining feature of pyroptosis [18,19]. GSDM-D is an executor of pyroptosis and belongs to the gasdermin (GSDM) protein family, which includes six paralogues: GSDM-A to GSDM-E, and PJVK (DFNB59). In their inactive forms, GSDM proteins are primarily localized in the cytoplasm. Upon activation, the N-terminal domains translocate to the plasma membrane to execute their pore-forming functions. The expression of GSDMs is tissue- and cell-type-specific. For instance, GSDM-A is expressed in the skin, GSDM-A/B/C in the gastrointestinal tract, and GSDMB/C in the lungs and reproductive organs. GSDM-D and GSDM-E, however, are ubiquitously expressed in immune cells such as macrophages and dendritic cells, highlighting their essential role in innate immunity [20].

IL-1β and IL-18 are pro-inflammatory cytokines that belong to the interleukin-1 protein family. Both are synthesized as inactive precursor forms which are converted to active forms upon cleavage by caspase-1 [21]. During canonical pyroptosis signaling, cell membrane rupture mediated by GSDM-D facilitates the release of IL-1b and IL-18, which promotes the recruitment of innate immune cells like neutrophils and macrophages and modulates the activation of adaptive immune cells like CD4^+^ T-cells or even CD8^+^ T-cells [22,23,24].

### 2.2. Noncanonical Pyroptosis Mediated by Caspase-4/5/11

Following the identification and characterization of the canonical pyroptosis pathway, multiple studies revealed that pyroptosis can also be activated through alternative signaling mechanisms, collectively referred to as noncanonical pathways. One such noncanonical pathway is triggered directly in response to the PAMP molecule LPS and involves the formation of a ‘noncanonical inflammasome’, distinct from the inflammasome generated via the NLRP3/ASC/caspase-1 axis (Figure 1B). In this noncanonical pathway, LPS binds directly, and with high affinity, to caspase-11 in mouse macrophages, or to its homologs, caspase-4 and caspase-5, in human monocytes, without the need for NLRP3-mediated PAMP sensing [25,26].

The binding of LPS to caspase-11 (or caspase-4/5 in humans) induces the formation of a “noncanonical inflammasome”, which is sufficient to induce the oligomerization and activation of these caspases [25]. In mouse macrophages, activated caspase-11 directly cleaves GSDM-D, resulting in cell membrane perforation and pyroptotic cell death. Although caspase-11 does not directly cleave pro-IL-1β or pro-IL-18, evidence suggests it can activate a cascade involving the NLRP3/caspase-1 inflammasome, ultimately leading to IL-1β processing and release [26,27]. However, the precise mechanisms by which caspase-11 interacts with the canonical inflammasome remain unclear.

### 2.3. Noncanonical Pyroptosis Mediated by Caspase-3/8

In addition to pyroptosis induction by caspase-1 or caspases-4/5/11, the apoptotic mediators caspase-3 and caspase-8 also have been found to play roles in inducing a noncanonical pathway of pyroptosis (Figure 1C). The gasdermin (GSDM) protein family comprises six members (GSDM-A, -B, -C, -D, -E, and PJVK). Cleavage of these proteins generates N-terminal fragments (N-GSDM) that form pores in the cell membrane, leading to pyroptotic cell death [28]. Recent studies have shown that GSDM-E is a specific substrate of caspase-3. In cells expressing GSDM-E, tumor necrosis factor (TNF) can induce caspase-3-mediated cleavage of GSDM-E into active N-GSDM-E fragments, which perforate the cell membrane and induce pyroptosis [29].

A role for caspase-8 in noncanonical pyroptosis has come to light in studies of caspase-1 and caspase-11 double knockout mice. Infection of macrophages from these mice with Legionella pneumophila triggers the recruitment of caspase-8 to a noncanonical inflammasome complex, which consists of neuronal apoptosis inhibitory protein 5 (NAIP5), NLR family CARD-containing protein 4 (NLRC4), and ASC. Formation of this complex results in the activation of caspase-8 and subsequent cell membrane perforation via a GSDM-D-independent process [30]. Key questions regarding this pathway that remain unanswered are as follows: (1) whether caspase-8 activation is necessary to trigger caspase-3-mediated GSDM-E activation; and (2) whether the absence of caspase-1/11 and GSDM-D is essential for caspase-8-mediated membrane rupture. Additional findings reveal complex roles for caspase-8 in inflammasome activity. One study reported that expression of a catalytically inactive caspase-8 mutant (C362S) leads to ASC inflammasome complex formation and results in caspase-1 activation and IL-1β secretion. Embryonic expression of this caspase-8 mutant causes necroptosis-independent embryonic lethality due to pyroptosis [31]. Another study found that Yersinia infection, by inhibiting TGF-β-activated kinase 1 (TAK1) or IKK kinases, induces cell death mediated by both GSDM-D and GSDM-E. This pathway operates independently of caspase-1 and caspase-11 but is dependent on caspase-8 activity [32]. While the role of caspase-8 in this process is significant, it is unclear whether caspase-8 directly cleaves GSDM proteins.

## 3. Inducing Pyroptosis in Cancer

The role of pyroptosis in cancer development is not fully understood. Pyroptosis occurs in innate immune cells (primarily macrophages and neutrophils) in response to pathogen invasion or cellular stress, leading to the release of cytokines that trigger an inflammatory defense. Chronic inflammation, however, can promote cancer initiation [33]. During cancer progression, pyroptosis-induced immune cell infiltration may contribute to both pro-tumor and anti-tumor effects, with the balance of interactions between cancer cells and immune cells playing a critical role in cancer outcomes.

Pyroptosis has been observed not only in immune and barrier epithelial cells but also in various cancer cell types, including those representing liver cancer [34], breast cancer [35], esophageal cancer [36], lung cancer [37], colon cancer [38], and cervical cancer [39]. Despite the complex nature of pyroptosis within the tumor immune microenvironment, the induction of pyroptosis in cancer cells may present a promising opportunity for cancer therapy. The induction of pyroptosis in cancer cells not only leads to direct cell death but also releases cancer antigens, which enable antigen-presenting cells (APCs) to present these antigens to immune cells, including CD8^+^ T-cells. Additionally, the secretion of inflammatory cytokines can further support anti-tumor immunity to some extent.

Here, we describe biomolecules that have been shown to induce pyroptosis in cancers and the potential for incorporating these molecules in new therapeutic approaches.

### 3.1. Chemotherapy Drugs That Induce Pyroptosis

Chemotherapy drugs are primarily known to induce cell death via caspase-3-mediated apoptosis. However, recent studies have shown that certain chemotherapeutic agents can also induce pyroptosis. In particular, topotecan, etoposide, cisplatin, and CPT-11 have been observed to trigger pyroptosis in SH-SY5Y neuroblastoma cells and MeWo skin melanoma cells, both of which exhibit high levels of gasdermin E (GSDM-E) expression [29]. These chemotherapeutic agents promote pyroptosis by inducing the caspase-3-mediated cleavage of GSDM-E. Generally, GSDM-E expression is low in most cancer cells, leading to apoptosis as the primary cell death pathway. However, in cancer cells with elevated GSDM-E expression, the cell death mechanism is shifted towards pyroptosis following chemotherapy treatment. This switch highlights the potential of GSDM-E as a key factor in modulating the response of certain cancers to chemotherapy [29]. As summarized in Table 1, multiple chemotherapy drugs have now been shown to induce pyroptosis in a variety of cancer types via caspase-3-mediated cleavage of GSDM-E.

### 3.2. Metformin

Metformin, well known as an anti-diabetic medication, has shown potential as an anti-cancer agent by inducing pyroptosis in cancer cells. Wang et al. [45] demonstrated that metformin induces GSDM-D-mediated pyroptosis in esophageal squamous cell carcinoma (ESCC) following inhibition of the oncogenic scaffold protein PELP1, a biomarker of poor prognosis. However, the mechanisms linking PELP1- and GSDM-D-mediated pyroptosis were not fully elaborated in this study.

Further research has demonstrated that metformin can promote cancer cell pyroptosis through activation of the SIRT1/NF-κB signaling pathway. Metformin activates SIRT1, an NAD^+^-dependent deacetylase involved in regulating immunity and inflammatory responses. SIRT1 activation enhances the expression of NF-κB (p65), which then promotes the induction of BAX and the activation of caspase-3, leading to GSDM-E-mediated pyroptosis. This cascade of events underscores metformin’s capacity to harness the SIRT1/NF-κB signaling axis to drive pyroptosis and positions metformin as a novel therapeutic option to induce pyroptotic cell death in cancer cells, particularly those resistant to traditional apoptotic pathways [46].

### 3.3. Small Molecule Inhibitors That Induce Pyroptosis

#### 3.3.1. DPP8/9 Inhibitors

Dipeptidyl peptidases (DPPs) are proteases that cleave the N-terminal dipeptide of their substrates. Inhibitors of DPP8 and DPP9 have attracted significant interest due to their ability to induce pyroptosis in immune cells [47]. DPP8/9 interacts with the inflammasome complex containing the PRR protein NLRP1 and CARD8 (caspase recruitment domain-containing 8 protein). When DPP8/9 binds to this inflammasome complex, CARD8 is retained within it. However, upon inhibition of DPP8/9, CARD8 is released from the complex, triggering caspase-1 activation and induction of pyroptosis [48]. The pyroptosis pathway induced by DPP8/9 inhibitors is primarily observed in monocytes and macrophages [47,49].

Several DPP8/9 inhibitors are currently available, including the nonselective DPP inhibitor Val-boroPro, the DPP8/9 selective inhibitor 1G244, as well as CQ31, and compounds 8j and L-allo-Ile-isoindoline [50,51,52,53]. Beyond immune cells, DPP8/9 inhibitors have been shown to induce pyroptosis in acute myeloid leukemia (AML) cell lines and primary AML samples [54]. While the effects of these inhibitors on solid tumors are not yet reported, DPP8 and DPP9 expressions have been detected in various normal tissues and a range of tumors, including brain tumors, gynecological malignancies, and liver cancer. Thus, DPP8/9 inhibitors represent a potential strategy for inducing pyroptotic cell death in solid cancers.

#### 3.3.2. BRD4 Inhibitor

BRD4 is a member of the bromodomain and extra-terminal domain (BET) protein family, and it binds to acetylated lysine residues on histone tails, thereby altering chromatin structure. The BRD4 inhibitor JQ1, a thienotriazolodiazepine-based small molecule, competitively inhibits BET-histone binding, preventing BET proteins from interacting with chromatin. Recently, JQ1 has been shown to induce pyroptosis in renal cell carcinoma (RCC) via the activation of NF-κB, which subsequently activates the NLRP3 inflammasome, leading to caspase-1-dependent pyroptosis [55]. While the precise interaction between BRD4 and NLRP3 remains unclear, another study reported that BRD4 is an essential regulator of NLRC4 inflammasome activation in Salmonella-infected bone marrow-derived macrophages [56].

#### 3.3.3. Other Inhibitors

Recent studies have demonstrated that inhibitors targeting EGFR, MEK, and ALK in lung cancer cells that express mutant forms of these proteins induce not only apoptosis but also pyroptosis. These inhibitors activate the intrinsic apoptotic pathway through the BIM-BAX-cytochrome c release signaling axis, which subsequently triggers caspase-3/GSDM-E-mediated pyroptosis. Notably, pyroptosis induced by these inhibitors occurs concurrently with caspase-3-mediated apoptosis, and there is a complex interplay among the proteins mediating both cell death pathways. However, the precise mechanisms underlying this crosstalk are incompletely understood [57].

In melanomas harboring the BRAF V600E/K mutation, the combined use of the BRAF inhibitor PLX4720 and the MEK inhibitor PD0325901 promotes GSDM-E cleavage and the release of HMGB1, along with an upregulation of IL-1β and IL-18, leading to pyroptosis induction [58]. This pyroptosis significantly enhances CD8^+^ T-cell infiltration into the tumor microenvironment, facilitating the immune-mediated inhibition of tumor growth. Interestingly, melanoma tumors that relapse after this combination therapy remain susceptible to etoposide- and doxorubicin-induced pyroptosis, highlighting the potential of pyroptosis induction as a therapeutic strategy not only for primary tumors but also for recurrent tumors resistant to targeted therapies [58].

Additional small molecule inhibitors that act to induce pyroptosis are summarized in Table 2.

### 3.4. Natural Products That Induce Pyroptosis

#### 3.4.1. Curcumin

Curcumin is a natural compound derived from the rhizomes of turmeric (*Curcuma longa*), a hydrophobic polyphenol (Table 3). The United States Food and Drug Administration (FDA) has approved the use of curcumin as a safe flavoring and antioxidant agent in certain foods [68]. Over the past two decades, extensive research has shown that curcumin interferes with multiple cell signaling pathways, impacting cancer by activating apoptosis, inhibiting proliferation, reducing inflammation, and suppressing angiogenesis and metastasis [69,70,71]. In light of its promising impact in preclinical cancer models, curcumin-containing products have been investigated in numerous clinical trials for treating pancreatic, breast, prostate, and colorectal cancers [72,73,74].

Recent studies indicate that curcumin can induce pyroptosis in cancer cells. In HepG2 hepatocellular carcinoma cells, curcumin treatment activates the intrinsic apoptosis pathway, which in turn triggers caspase-3/GSDM-E-mediated pyroptotic cell death. This process results from elevation in the levels of reactive oxygen species (ROS) following curcumin treatment [75]. However, curcumin is far from a specific inducer of pyroptosis, as it also induces other forms of cell death, including apoptosis, necroptosis, and ferroptosis [86,87].

#### 3.4.2. Iron

Iron plays a crucial role in both the formation and destruction of intracellular ROS, which serve as key mediators in multiple forms of cell death. Mitochondrial ROS, for example, drives intrinsic apoptosis, while ROS are also integral to necroptosis, ferroptosis, and, as recently identified, ROS-induced pyroptosis [88]. In melanoma cells, the combination of iron with the ROS-inducing agent carbonyl cyanide m-chlorophenyl hydrazone (CCCP) markedly amplifies ROS signaling, resulting in oxidation and oligomerization of the mitochondrial outer membrane protein TOM20. Oxidized TOM20 subsequently recruits BAX to the mitochondria, facilitating cytochrome c release into the cytosol and caspase-3 activation, which then leads to GSDM-E-mediated pyroptosis, highlighting a mechanistic pathway by which intracellular iron and enhanced ROS drive pyroptosis in melanoma [89].

#### 3.4.3. Cucurbitacin B

Cucurbitacin B (CuB) is a bioactive compound derived from *cucurbitaceae* plants that has shown significant promise as an anti-tumor and anti-inflammatory agent. In non-small cell lung cancer (NSCLC), cucurbitacin B induces pyroptosis by binding directly to Toll-like receptor 4 (TLR4). This binding activates the NLRP3 inflammasome, resulting in the cleavage of GSDM-D and subsequent pyroptotic cell death. Cucurbitacin B further promotes pyroptosis by increasing the production of mitochondrial ROS, leading to the accumulation/oxidation of mitochondrial TOM20, as well as to the release of cytosolic calcium. In mouse models of NSCLC, cucurbitacin B effectively inhibits tumor growth and shows superior anti-tumor effects compared to standard treatments, underscoring its potential as a targeted therapy through the TLR4/NLRP3/GSDMD signaling axis [90].

Beyond its anti-cancer role, cucurbitacin B also exhibits anti-inflammatory properties, particularly in osteoarthritis. In osteoarthritis models, cucurbitacin B interacts with key signaling proteins, including NRF2 and NF-κB, to inhibit the secretion of pro-inflammatory cytokines such as IL-1β and IL-18, thereby reducing inflammation [91]. These effects indicate that the impact of cucurbitacin B treatment may differ according to tissue and disease type, acting as an anti-inflammatory agent in osteoarthritis and triggering pyroptosis in cancer. This adaptability makes cucurbitacin B a strong candidate for distinct therapeutic applications across multiple diseases settings.

Other natural products that induce pyroptosis are summarized in Table 3.

### 3.5. Pathogen or Pathogen-Derived Compounds That Induce Pyroptosis

#### Lipopolysaccharide

Lipopolysaccharide (LPS) is a bacterial endotoxin known to trigger pyroptosis in immune cells, particularly in monocytes and macrophages. In HT29 colon cancer cells, LPS exposure induces cleavage/activation of GSDM-D, leading to the secretion of IL-1β and IL-18 and, ultimately, pyroptotic cell death. Furthermore, LPS has been shown to increase the chemosensitivity of HT29 cells to oxaliplatin, and this combined treatment enhances GSDM-D-mediated pyroptosis. These findings suggest that combination of LPS with chemotherapy drugs may provide a strategy to promote pyroptosis and improve therapeutic outcomes in colon cancer [92].

For therapeutic purposes focused on the induction of pyroptosis, researchers have utilized bacterial outer membrane vesicles (OMVs) as natural carriers to deliver LPS into cancer cells. OMV-LPS has been shown to effectively induce GSDM-D-mediated pyroptosis in cancer cells, leading to a significant inhibition of tumor growth in vivo [93]. Additionally, OMV-LPS treatment promoted greater CD8^+^ T-cell infiltration into the tumor microenvironment, enhancing anti-tumor immunity. This approach represents a promising strategy for treating solid tumors by inducing cancer cell death and stimulating immune responses [93].

Other pathogen or pathogen-derived components that induce pyroptosis in cancer cells are summarized in Table 4.

### 3.6. Endogenous Metabolites That Induce Pyroptosis

Metabolites play pivotal roles in cancer development, influencing cellular processes like proliferation, survival, and immune evasion within the tumor microenvironment. Recently, several endogenous metabolites—including omega-3 docosahexaenoic acid, citric acid, α, β-unsaturated ketone, and α-ketoglutarate (α-KG)—were identified as inducers of pyroptosis (Table 5). These metabolites can engage inflammasome pathways or enhance ROS production, thereby activating pyroptotic signaling in cancer cells.

As an example, α-KG is a key metabolite in the tricarboxylic acid (TCA) cycle and is involved in lipid biosynthesis, oxidative stress response, protein modification, autophagy, and cell death. α-KG induces pyroptosis in cancer cells via caspase-8-mediated cleavage of GSDM-C, showing promise for limiting tumor growth and metastasis. Treatment with DM-α-KG, a cell-permeable α-KG derivative, elevates ROS levels, which oxidizes and triggers endocytosis of the plasma membrane receptor DR6. This process recruits pro-caspase-8 and GSDM-C into a “receptosome”, where active caspase-8 cleaves GSDM-C, initiating pyroptosis [103]. This mechanism relies on an acidic environment, where α-KG is converted to L-2-hydroxyglutarate (L-2HG) by malate dehydrogenase 1 (MDH1), boosting ROS production. The presence of lactic acid further intensifies this effect, sensitizing even pyroptosis-resistant cancer cells to α-KG-induced pyroptosis [103]. Together, these insights reveal the dual role of metabolites as drivers of tumor progression and as therapeutic agents. Further exploration into metabolite-driven pyroptosis could inspire innovative treatments that reshape the tumor immune microenvironment for more effective cancer therapy.

## 4. Challenges and Opportunities

The induction of pyroptosis shows great promise as a therapeutic strategy, especially for cancers resistant to conventional chemotherapies that induce apoptosis. Chemotherapy-induced cell death involves not only apoptosis but also necroptosis and pyroptosis, yet clear molecular mechanisms that differentiate these processes or highlight crosstalk are not well defined. Numerous chemotherapeutic agents trigger the intrinsic apoptotic pathway through caspase-3 activation. This, in turn, leads to the cleavage of multiple substrates, such as PARP-1, which promote apoptotic cell death, and GSDM-E, which initiates pyroptosis. Although apoptosis and pyroptosis may occur simultaneously during caspase-3 activation, pyroptosis is more immunogenic and promotes immune cell infiltration, particularly by CD8^+^ T-cells, thereby boosting anti-tumor responses. Nonetheless, further research is necessary to clarify how apoptosis might be switched toward pyroptosis to enhance therapeutic outcomes.

The role of pyroptosis in various stages of cancer development is still not fully understood. While chronic inflammation can facilitate cellular transformation, cytokine release due to pyroptosis has the potential to attract effector immune cells to combat cancer. A significant challenge is how to effectively induce pyroptosis in cancers with varied genetic backgrounds. For instance, many cancer cells demonstrate low GSDM-E expression, making pyroptosis induction more challenging. Overcoming this limitation requires a deeper understanding of how pyroptosis affects cancer progression across different genetic contexts and tumor types. To date, no clinical trials have been undertaken with the primary goal of targeting pyroptosis in cancer. Combined therapies that utilize agents capable of converting apoptosis into pyroptosis may enhance treatment efficacy, particularly in apoptosis-resistant cancers. Pyroptosis facilitates the conversion of immunologically “cold” tumors into more immunogenic phenotypes, thereby amplifying the advantages of immunotherapy. Merging pyroptosis-inducing strategies with immunotherapeutic methods might significantly advance cancer treatment. It is crucial to comprehend the mechanisms that regulate pyroptosis and its interplay with the immune microenvironment to advance this strategy. Since pyroptosis-induced inflammation may, under certain circumstances, contribute to tumor progression, combining anti-inflammatory drugs may serve as a complementary approach to enhance the efficacy of anti-pyroptosis strategies. This combinatorial approach could help mitigate the pro-tumorigenic effects of inflammation while simultaneously targeting pyroptosis, offering a more comprehensive therapeutic strategy for tumor suppression. By tackling these challenges, pyroptosis-based therapies may become vital for innovative cancer treatments, providing new hope for patients with resistant tumors.

Natural products, primarily derived from plants and microbes, have proven to be valuable sources of anti-cancer agents, including those capable of inducing pyroptosis. These compounds enhance ROS generation and activate pathways comprising signaling molecules like NLRP3, MEK/ERK1/2, and JNK, thereby promoting tumor cell death while fostering an immune-stimulatory tumor microenvironment. However, limitations such as low bioavailability and off-target effects have hindered the clinical application of natural products. Nanotechnology offers a potential solution by improving the solubility, stability, and specificity of natural products through nanosizing and targeted delivery systems. The incorporation of pyroptosis-inducing agents into nanoparticles enhances their efficacy and enables synergy with therapies like chemotherapy and immunotherapy. These advancements position natural product-based pyroptosis inducers as promising tools for precise and potent cancer immunotherapy.

Collectively, despite significant progress, challenges remain in translating pyroptosis-based therapies to clinical settings. Effective screening strategies are essential for identifying potent pyroptosis-inducing agents. Addressing tumor heterogeneity and the pro-inflammatory nature of pyroptosis is also critical to minimizing side effects and increasing treatment specificity. By integrating pyroptosis inducers with other therapeutic modalities, these combinatorial strategies may overcome treatment resistance, stimulate robust immune responses, and establish pyroptosis as a cornerstone in the fight against cancer.

Cancer cells exhibit unique vulnerabilities to cell death, and exploitation of these vulnerabilities has strong potential to lead to tumor-selective anti-cancer therapies. Pyroptosis represents a new area of cell death research that should be explored as a means of provoking cell death in cancer cells and tumors. At present, only a modest number of biomolecules have been identified to promote pyroptosis. More work is needed to identify pyroptosis-inducing agents, and combination of these agents with immune modulating therapies is warranted. Coincident with these investigations, it will be important to closely monitor whether selective induction of pyroptosis leads to adverse toxicities, and whether biomarkers that will help determine which patient populations are most likely to benefit from treatment with pyroptosis inducers can be identified. As research into these questions continues, we will learn the full utility of targeting pyroptosis to improve clinical outcomes in patients suffering from cancer.

## Figures and Tables

**Table 1:** Chemotherapy drugs that induce pyroptosis.

Reagents	Category	Mechanism	Cells	Reference
Topotecan	Topoisomerase 1 inhibitor	caspase-3/GSDM-E	SH-SY5Y, MeWo	[29]
Etoposide	Topoisomerase II inhibitor	caspase-3/GSDM-E	SH-SY5Y, MeWo	[29]
Cisplatin	Alkylating agent	caspase-3/GSDM-E	SH-SY5Y, MeWo, A549	[37,40]
5-Fu	Anti-metabolites	caspase-3/GSDM-E	SGC-7901 MKN-45	[41]
Paclitaxel	Microtubules interference	caspase-3/GSDM-E	A549	[41]
Ru (II) polypyridyl + Paclitaxel		caspase-1/GSDM-D	HeLa	[42]
Lobaplatin	Platinum	caspase-3/GSDM-E	HT-29, HCT116	[38]
Doxorubicin	Anthracycline	caspase-3/GSDM-E	HepG2, Hep 3B	[43]
FL118	Camptothecin	_	Colon cancer cells	[44]

**Table 2:** Small-molecule inhibitors that induce pyroptosis.

Name	Target	Mechanisms	Cancer Types	Reference
BI2536	PLK1	caspase-3/GSDM-E	Esophageal	[59,60]
Alpha-NETA	Choline acetylcholine transferase	caspase-4/GSDM-D	Ovarian	[61]
BIX-01294 (BIX)	G9a	caspase-3/GSDM-E	Gastric	[62]
NO. 0449-0145	APE1	caspase-4/GSDM-D	Lung	[63]
Famotidine	Histamine H2-receptor	GSDM-E	gastric	[64]
Bexarotene	RXR	caspase-4/GSDM-E	ovarian	[65]
AT7519	CDK	caspase-3/GSDM-E	Glioblastoma	[66]
Elraglusib	GSK-3	GSDM-B	Colon	[67]

**Table 3:** Natural products that induce pyroptosis.

Name	Category	Mechanisms	Cancer Types	Reference
Curcumin	Polyphenol	caspase-3/GSDM-E	Liver	[75]
Anthocyanins	Flavonoid	caspase-1/GSDM-D	Oral	[76]
Miltirone	Abietane-type diterpenoids	caspase-3/GSDM-E	Liver	[77]
Tetraarsenic hexoxide	Arsenic oxide	caspase-3/GSDM-E	Breast	[78]
Triptolide	Diterpenoid epoxide	caspase-3/GSDM-E	Oral	[79]
Trichosanthin	Ribosome-inactivating protein	caspase-1/GSDM-D	Lung	[80]
Aloe-emodin	Anthraquinone	caspase-3/GSDM-E	Cervical	[81]
Myricetin	Flavonoid	caspase-3/GSDM-E	Lung	[82]
Luteolin	Flavonoid	caspase-1/GSDM-D	Colon	[83]
Mallotucin D	Flavonoid glycoside	caspase-3/GSDM-E	Liver	[84]
Shikonin	Naphthoquinone	caspase-3/GSDM-E	Gastric	[85]

**Table 4:** Pathogen or pathogen-derived compound that induce pyroptosis.

Name	Category	Mechanisms	Cancer Types	Reference
4-hydroxybenzoic acid	Benzoic acid derivatives	caspase-1/GSDM-D	Lung	[94]
EV-A71	Enterovirus	caspase-1/GSDM-D	Neuroblastoma	[95]
CagA	Protein from *Helicobacter pylori*	caspase-1/GSDM-D	Gastric	[96]
Apoptin	Protein from chicken anemia virus	caspase-3/GSDM-E	Colon	[97]
Talaromyces marneffei	Fungus	caspase-1/GSDM-D	Liver	[98]
Coxsackievirus group B3	Enterovirus	caspase-3/GSDM-E	Colon	[99]

**Table 5:** Endogenous metabolites that induce pyroptosis.

Name	Category	Mechanisms	Cancer Types	Reference
Omega-3 docosahexaenoic acid	Polyunsaturated fatty acid	caspase-1/GSDM-D	Breast	[100]
Citric acid	Tricarboxylic acid	caspase-4/GSDM-D	Ovarian	[101]
α, β-Unsaturated ketone	Unsaturated carbonyl compound	caspase-3/GSDM-E	Lung cancer	[102]

## Data Availability

Not applicable.
